# Ultra Low-Dose Radiation: Stress Responses and Impacts Using Rice as a Grass Model

**DOI:** 10.3390/ijms10031215

**Published:** 2009-03-16

**Authors:** Randeep Rakwal, Ganesh Kumar Agrawal, Junko Shibato, Tetsuji Imanaka, Satoshi Fukutani, Shigeru Tamogami, Satoru Endo, Sarata Kumar Sahoo, Yoshinori Masuo, Shinzo Kimura

**Affiliations:** 1 Health Technology Research Center, National Institute of Advanced Industrial Science and Technology (AIST), West, Tsukuba, Ibaraki 305–8569, Japan; E-Mails: rjunko@nifty.com (J.S.); y-masuo@aist.go.jp (Y.M.); 2 Research Laboratory for Biotechnology and Biochemistry (RLABB), GPO Box 8207, Kathmandu, Nepal; E-Mails: plantproteomics@gmail.com (R.R.); gkagrawal123@gmail.com (G.K.A.); 3 Research Reactor Institute, Kyoto University (KURRI), Osaka, Japan; E-Mails: imanaka@rri.kyoto-u.ac.jp (T.I.); fukutani@rri.kyoto-u.ac.jp (S.F.); 4 Laboratory of Growth Regulation Chemistry, Akita Prefectural University, Akita 010–0195, Japan; E-Mail: tamo_chem@akita-pu.ac.jp; 5 Graduate School of Engineering, Hiroshima University (HU), Hiroshima, Japan; E-Mail: endos@hiroshima-u.ac.jp; 6 Research Center for Radiation Protection, National Institute of Radiological Sciences (NIRS), Chiba 263–8555, Japan; E-Mail: sahoo@nirs.go.jp; 7 Department of Research Planning and Coordination, Japan NIOSH, Kawasaki, Japan; E-Mail: kimura@h.jniosh.go.jp

**Keywords:** Contaminated soil, γ-ray, Marker genes, *Oryza sativa*, Phytoalexins

## Abstract

We report molecular changes in leaves of rice plants (*Oryza sativa* L. - reference crop plant and grass model) exposed to ultra low-dose ionizing radiation, first using contaminated soil from the exclusion zone around Chernobyl reactor site. Results revealed induction of stress-related marker genes (Northern blot) and secondary metabolites (LC-MS/MS) in irradiated leaf segments over appropriate control. Second, employing the same *in vitro* model system, we replicated results of the first experiment using in-house fabricated sources of ultra low-dose gamma (γ) rays and selected marker genes by RT-PCR. Results suggest the usefulness of the rice model in studying ultra low-dose radiation response/s.

## 1. Introduction

It is now over 20 years since the Chernobyl nuclear accident, and the resulting environmental impact of radiation on living organisms that followed is still being studied [1]. The initial 2800 km^2^ radius exclusion zone around the Chernobyl reactor site where radioactive fallout heavily contaminated the environment was later modified and extended to cover 4,300 km^2^. The aftereffects are still being felt in Southern Belarus, where the levels of cesium-137 (^137^Cs) and strontium-90 (^90^Sr) – both fission products from the reactor core – are and will remain high for decades to come. Other than human life directly or indirectly affected by radiation the plant life is also continuously affected, being unable to move out of the hot zone. The soil carries most of the radio-contamination, although use of fertilization and liming of the fields has been used to expel or bind the radionuclides. Nevertheless, a low to high level of contamination still exists in and around areas surrounding the Chernobyl nuclear reactor site.

With this background, the radioactively contaminated soil should provide us with a good source material for studying the effects of ionizing radiation (IR) on living organisms. Indeed, a single study in two genetically identical wheat populations revealed a remarkably strong induction of germline mutation upon chronic exposure to low-dose IR (0.3 Gy over a period of 10 months) from the Chernobyl accident [2]. Various physiological factors for the observed increased frequency of heterozygous structural variants in the offspring derived from exposed plants were considered before reaching to a conclusion that heterozygous variation was probably due to a radiation-induced increases in microsatellite mutation in the exposed plants. The results itself surprised Kovalchuk and coworkers, and we quote them – theoretically, this low-level exposure should not cause such a large increase in the mutation rate, suggesting that chronic exposure to IR has effects as yet unknown [2]. This one report highlighted the effect of low doses of IR in causing plant stress response. Therefore, the use of plants to study low- or ultra low-dose IR becomes a reality, and which is communicated in this report.

The plant model used was rice (*Oryza sativa* L.) – a grass plant, reference monocot plant, and potential human model. Why rice? In previous extensive studies, our and other research groups have been examining the effects of a wide variety of abiotic and biotic stresses in rice (for a review see [3]) primarily because its genome is sequenced. Moreover, rice is also a critical food crop for almost half the human population. With its sequenced genome rice has been termed the Rosetta Stone (by Dr. Robin Buell, a lead investigator for The Institute for Genomic Research, TIGR) and a cornerstone for cereal crop functional genomics by our group [4]. During the past 3 years, Rakwal and coworkers (at AIST, RLABB, NIRS, KURRI, HU and Japan NIOSH) have extended the on-going examination of the stress responses against a wide variety of environmental stimuli to the relatively unstudied IR [including gamma (γ)-, X-rays, and neutrons, and heavy ions] in rice. For this the established two-week-old rice seedling *in vitro* model system [3,5,6] is being followed. In this study, ongoing in stages since the year 2005, we examined the effects of ultra low-dose radiation in rice leaves by observing the changes in selected marker genes and metabolites related to the defense/stress response pathways against radioactively contaminated Chernobyl soil (CCS), and γ-rays. Our present study has showed that ultra low-dose radiation elicits a defense/stress response in rice cells, which is a novel finding. More importantly, it suggests the possibility of using rice plant as a simple and good model for investigating the responses to IR, including the relatively uninvestigated ultra low-doses.

## 2. Experimental Section

### 2.1. Soil sample and gamma- and beta- ray measurements

The radioactive CCS was collected during an on-site visit to Masany (Belarus; the site is 10 Km from the Chernobyl nuclear reactor) by one of the co-authors, Dr. Shinzo Kimura, in November of 2002. The γ-ray measurement was performed on 20 g of contaminated soil before the decomposition of samples, using a Ge-detector (CANBERRA GC1318, Connecticut, USA; 1.9 keV half bandwidth), coupled to a multichannel analyzer (Laboratory Equipment Co., MCA-48F, Ibaraki, Japan) using standard procedures. During measurement, one channel was set to 0.5 keV so that measurement up to 4,000 keV was possible. The available ^90^Sr (β-ray emission spectra) in 1 g of contaminated soil was measured by a low background beta-ray spectrometer (LBC-471Q, ALOKA Co. LTD, Tokyo Japan) according to standard procedures. For the in-house radiation experiment, the γ-ray exposure rate emitted from the fabricated disc shaped radiation sources was measured using glass fluorescence dosimeters (GD-301, Dose Ace FGD-1000, ALOKA) under the same conditions as the experiment using the same glass Petri dishes (Supplementary Figure 1).

### 2.2. Test plants and in vitro rice seedling system

Rice (*O. sativa* L. cv. Nipponbare) seedlings were grown for two-weeks (after germination) under white fluorescent light (wavelength 390–500 nm, 150 mmol·m^−2^·s^−1^, 12 h photoperiod) at 25°C and 70% relative humidity [5,6]. For treatments, the middle portions of fully expanded leaves (approximately 2 cm long segments cut with clean scissors) were placed on 25 mL MQ™ water (Millipore, Waters) in clean Petri dishes, which were subsequently placed over 20 g CCS contained in the cover of the same Petri dish, and incubated in the growth cabinet under continuous light (150 mmol·m^−2^·s^−1^). There was no direct connection between the soil and the leaf segments, which were separated by the water in the Petri dish. Leaf segments floated on MQ water and placed over uncontaminated soil served as an appropriate control (CON). Sixteen individual leaf segments were sampled and pooled (from two independent experiments performed in the year 2005) at the times indicated and immediately frozen in liquid nitrogen and stored at −80°C. Leaf segments (at 96 h) were also photographed with a digital camera. In the second experiment using in-house fabricated radiation doses of ultra-low γ-ray, rice plants were grown exactly as described above and leaf segments were also prepared for irradiation in exactly the same way. Following irradiation, the leaf segments (ca. 16 per Petri dish) were pooled and immediately immersed in liquid nitrogen. A dummy experiment using cut leaf segments without γ-ray (control, C: 5.0 ± 0.4 μGy/3 days) in the same growth cabinet as the treatments, along with leaves from whole plants (WP) kept in the experimental room for 3 days (background: 4.5 ± 0.2 μGy/3 days) was also sampled. The positive control (WP) at 3 days was taken by cutting the rice leaves in exactly the same manner as on day 1, followed by immediate freezing in liquid nitrogen. For time-course experiment, another set of rice plants were cultivated as above, and then rice leaves were cut into segments and treated with a dummy (no radiation source) or ultra low-dose γ-ray for 1 to 72 h. These experiments (γ-irradiation) were performed four times at different periods of the year 2008 and in the beginning of 2009.

### 2.3. Total RNA extraction and Northern analysis

Total RNA isolation and Northern blot analysis was performed as described previously [5,6]. Briefly, deep frozen leaf segments were ground to a very fine powder in liquid nitrogen followed by extraction of total RNA with QIAGEN RNeasy Plant Maxi Kit (QIAGEN, Maryland, USA). The quality and equal quantity of the RNA was determined spectrophotometrically (NanoDrop, Wilmington, Delaware, USA) and visually confirmed using formaldehyde-agarose gel electrophoresis. Gene specific cDNA probes for rice: PR genes, *OsPR1b* (basic, accession number U89895), *OsPR5* (thaumatin-like protein, accession number X68197) and *OsPR10a* (accession number D38170); OB-related genes, *OsCATc* (catalase, accession number AB020502), *OsPOX* (peroxidase, accession number X66125) and *OsAPX1/2* (ascorbate peroxidase, accession numbers AB053297/BAB20889); SM-related genes, *OsPAL* (phenylalanine lyase, accession number X87946) and *OsCHS* (chalcone synthase, accession number X89859); P-related genes, *OsRbsL* (ribulose-1,5-bisphosphate carboxylase oxygenase, RuBisCO large subunit; [7]), *OsRbsS* (RuBisCO small subunit; [7]) and *OsCab* (chlorophyll *a/b* binding protein; [7]). Membranes were washed with 2 × SSC and 0.1% (w/v) SDS at 65 °C for 1 h, and exposed to an X-ray film (Kodak, Tokyo, Japan) using two intensifying screens for 2 d at −80°C. Rice *actin* gene was used as a control. Equal loading (20 μg) of total RNA was also confirmed by staining the blotted membrane with methylene blue (rRNA).

### 2.4. Semi-quantitative RT-PCR analysis

**Figure 1.**
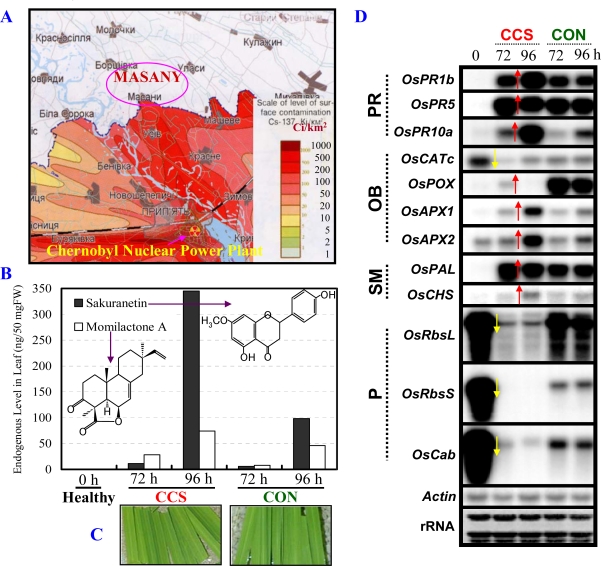
The effect of Contaminated Chernobyl Soil (CCS) on rice leaf biology. (A) Soil was collected during an on-site visit to Masany. (B) Levels of sakuranetin and momilactone A increase in leaves at 72 and 96 h after exposure (5.34 μGy/day) to CCS over control (CON), compared to non-detection in healthy leaves (0 h) at the start of the experiment. (C) Photograph (Digital Camera, Sony Cyber-shot 5.0 Megapixels) of representative leaf segments 96 h after exposure to CCS. (D) Northern blot analysis reveals changes in mRNA level of marker genes at 72 and 96 h using gene specific cDNA probes for rice genes: *OsPR1b, OsPR5* and *OsPR10a; OsCATc, OsPOX* and *OsAPX1/2; OsPAL* and *OsCHS;, OsRbsL, OsRbsS* and *OsCab.* Leaf segments cut from the 3^rd^/4^th^ leaves of two-week-old seedlings were used as the experimental material (see also Section 2.2 for details).

Total RNA was extracted as described above for subsequent RT-PCR analysis with eight selected “marker” genes based on the first experiment using CCS (see Figure 1). First-strand cDNA was synthesized in a 50 μL reaction mixture with a StartaScript™ RT-PCR Kit (Stratagene) using 10 μg total RNA isolated from leaves of irradiated and control plants. Specific primers were designed from the 3′-UTR regions (forward and reverse primer sequences are provided in Supplementary Table 1) of each of the genes used in this study by comparison and alignment with all available related genes in the databases, NCBI and KOME (Knowledge-based Oryza Molecular biological Encyclopedia, http://cdna01.dna.affrc.go.jp/cDNA/). The RT-PCR was carried out exactly as described [8].

### 2.5. Phytoalexin estimation

Sakuranetin and momilactone A in leaves were analyzed and estimated using standards by a liquid chromatography tandem mass spectrometry technique as previously established and described [9].

## 3. Results and Discussion

### 3.1. Radioactive Chernobyl soil triggers defense/stress responses in rice leaves

Measurements of radioactivity revealed that the CCS emits radiation, which is due to the fact that it consists of long-lived radioisotopes ^137^Cs and ^90^Sr as fission products (Figure 1A). Measurements on CCS showed radiation level to be 5.34 μGy/day (Table 1). This dose is ca. 10.90-fold over the (shielded) control level indicating an extremely low (ultra) level of emitted radiation.

Examination of rice stress-inducible molecular markers in the rice leaves exposed to CCS first revealed strong accumulation of two major rice phytoalexins (sakuranetin and momilactone A) at 72 and 96 h post-exposure (Figure 1B). The phytoalexins are prominent and established secondary metabolite (SM) markers commonly used to investigate stress responses in rice [reviewed in 3]. A slight increase in these phytoalexins in the control (CON) may be part of a stress response triggered by continuous light exposure. Nonetheless, a strong induction of phytoalexins is also seen upon pathogen attack and other abiotic stresses such as exposure to ozone [reviewed in [3], indicating a conservation in the induction of these secondary metabolites among numerous abiotic stresses, including to ultra low-dose IR. There also appeared to be a change in leaf color after exposure to CCS. At 96 h post-exposure leaf segments showed subtle change in color from dark green (CON) to light green (CCS), suggesting a possible damage to the photosynthetic apparatus (Figure 1C). In the case of ozone, such damage also correlated well with phytoalexins accumulation (reviewed in [3]).

Secondly, northern blot analysis revealed distinct changes in the mRNA expression of specific maker genes in CCS over CON (Figure 1D). The induction (red arrows) or suppression (yellow arrows) of these genes is marked for clarity. Of note, these genes were selected based on our studies of the rice defense/stress responses (reviewed in [3]). These prominently included the pathogenesis-related (PR) genes *OsPR1b*, *OsPR5*, and *OsPR10a*. The oxidative burst (OB)- (*OsCATc*, *OsPOX*, *OsAPX1/2*), SM- *(OsPAL* and *OsCHS),* and photosynthesis (P)- *(OsRbsL, OsRbsS,* and *OsCab)* related genes also showed changed expression profiles. A strong induction of the three PR gene transcripts in CCS over CON suggests radiation from CCS causes an activation of the defense/stress response pathway. On the other hand, a differential (induction or suppression) response of the antioxidant-related transcripts by CCS exposure was observed. The *OsCATc* transcript is strongly suppressed by CCS (at 72 h), whereas, the *OsAPX1* and *2* transcripts are strongly induced at 96 h over CON. On the other hand, the *OsPOX* transcript was weakly induced at 72 h in CCS over the potent induction of its transcript in CON at both times points. This result suggests that the genes responsible for antioxidant defense may be modulated by the cellular levels of reactive oxygen species (ROS) upon radiation. In future studies, the type of ROS and their levels will have to be measured and compared in irradiated and control samples to clarify this point. Moreover, as expected the P-related *OsRbsL, OsRbsS,* and *OsCab* transcripts, the best studied examples of plant nuclear encoded genes [10,11], were found to be strongly suppressed by exposure to CCS. This might relate to the observed loss of green color in irradiated leaves (Figure 1C). Damage to the photosynthesis may also lead to an unbalanced ROS level leading to the differential modulation of the OB-related gene expressions seen in Figure 1C. This Northern blot analysis data are also in good agreement with a preliminary DNA microarray analysis for rice leaf segments exposed to CCS, which showed large changes in gene expressions in defense/stress-related categories [12].

Differential expression of marker metabolites and genes in leaves upon CCS exposure suggest a subtle effect of ultra low-dose of radiation in rice cells. The study of Kovalchuk and co-workers shows the rate of mutation by long-term internal and external exposure from radiation in wheat [2]. However, we carried out a study limited to external short-term ultra low-dose irradiation exposure, in order to clarify the effect of radiation by observing changes of biological active metabolites and genes. Therefore, our rice *in vitro* model system is indicative of an active reaction triggered by ultra low-level radiation in the living cells.

### 3.2. Ultra low-dose gamma (γ)-radiation also triggers the expression of defense/stress marker genes

As this striking observation detailed above (Figure 1) mainly due to γ -ray from CCS was never reported before, we further asked a simple question: whether ultra low-dose γ-radiation mimics the observed responses? To investigate this, we designed an in-house experiment where a disc-shaped γ-ray ^137^Cs source was fabricated to irradiate leaf segments in a similar manner as done with CCS. The experiment was designed to perform six γ-radiation doses (13 ± 1, 25 ± 2, 45 ± 2, 110 ± 10, 190 ± 10 and 380 ± 20 μGy/3 days) in parallel to appropriate control as illustrated and described in Supplementary Figure 1.

**Figure 2.**
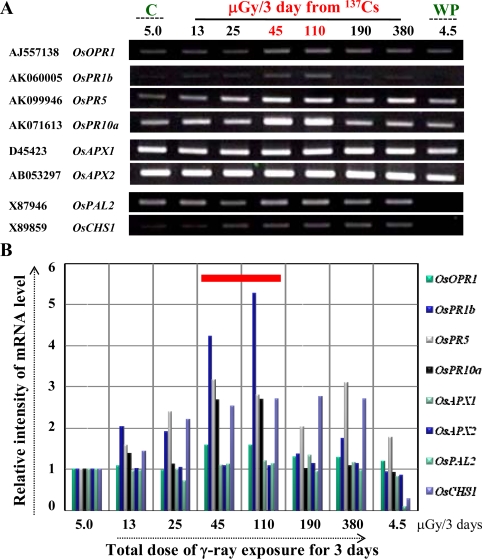
Effects of ultra low-dose γ-radiation using in-house fabricated radiation source on mRNA expression level of marker genes in leaves of rice seedlings by RT-PCR. (A) Leaf segments were prepared as described in Figure 1, pooled together after treatment for total RNA isolation followed by quality check, cDNA synthesis, and RT-PCR analysis. The gene specific primers used are provided in Supplementary Table 1. Leaf segments were also sampled from dummy (C: control), and whole plants (WP: whole plant control) that served as a positive control for background radiation in the room where the experiment (in an incubator) was carried out. (B) The relative intensity of mRNA level of each transcript in (A) are presented. The intensities were calculated by the ATTO image analysis software (ATTO, Tokyo, Japan). Results are the representative of two independent experiments.

Using semi-quantitative RT-PCR, we found that ultra low-dose γ-radiation (2.6-times the background) activated the expression of classical marker genes at mRNA level for stress responses in plants associated with jasmonic acid biosynthesis *(OsOPR1),* and the defense/stress-related [PR *(OsPR1b, OsPR5, OsPR10a),* OB *(OsAPX1, OsAPX2*), and SM *(OsPAL2, OsCHS1)]* pathways (Figure 2A). The relative intensity of mRNA level is presented graphically for clarity (Figure 2B). Interestingly, we observed that the induction of these marker genes was dependent on the applied dose of ultra low-dose γ-ray, specifically and strikingly in the dose range of 45 to 110 μGy/3 days (red bar in Figure 2B). This result suggests a potential threshold for ultra low-dose in triggering a defense/stress response, which nevertheless remains to be further investigated. Nonetheless, these results indicate that ultra low-dose γ-radiation mimics the stress-related responses observed initially with CCS, where all these genes were dramatically changed at the mRNA level along with phytoalexins accumulation (see [12] and Figure 1).

**Figure 3.**
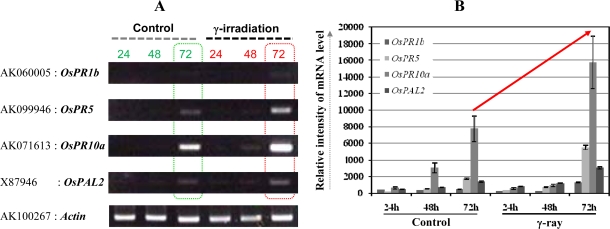
Ultra low-dose γ-irradiation induces strong expression of the PR-related defense/stress markers. The dummy (Control, dose rate of 5 μGy/3 day) and irradiated (dose rate of 110 μGy/3 day) samples were prepared for total RNA extraction and RT-PCR (A) as described in sections 2.3 and 2.4. In A, dotted boxed rectangles in green and red highlight the differential gene expression levels between control and treatment. On the right-hand side, the relative intensity of mRNA level of each gene is presented (B). The upward pointing red arrow shows the drastic change in rice marker gene expressions upon treatment, in particular the distinct increase in expression of the *OsPR10a* gene transcript. Actin gene expression was also used as a loading control (data not shown). Results are the representative of two independent experiments.

There were some questions from the non-biologists (physicists) in this collaborative study – How does cut influence the expression of genes at mRNA level in the leaf segments? Is it not necessary to check gene expression patterns for the genes used in Figure 2 at early time-points to check their stability, specificity and also the reproducibility? The above questions were a valid argument deserving attention and answer to how the gene expression unravels at early time points till 72 h. Moreover, as northern blotting and RT-PCR are two different techniques it was necessary to dispel any doubts on the validity of the RT-PCR approach for our physicist colleagues. Therefore, a new set of rice plants were grown, and a time-course experiment was performed using the 110 μGy/3 days as an example and time points of 1 to 72 h for control and 24 to 72 h for γ-ray (Figure 3, and see Supplementary Figure 2). The reason for doing a detailed time-course sampling for control was to show that these genes selected here do not express early just by cutting the leaf but are (late) response genes (Supplementary Figure 2). Of note, the cut-induced response in rice leaves has been well documented and the *in vitro* model system well established to study the molecular responses against numerous external stimuli including wounding by cut [reviewed in 3 and references therein]. Results showed a clear and strong induction of the four PR genes at 72 h by γ-ray over control (Figure 3). This clearly indicated that gene expression changes were due to γ -radiation and not by wounding where a considerably weaker expression was seen. Moreover, the results were reproducible (especially at the 72 h period, which was repeatedly analyzed) between these two independent experiments performed in 2008 and 2009, respectively (Figures 2 and 3). Furthermore, the results strongly support our northern blot analysis data from the first independent set of experiments performed in 2005 (Figure 1D).

### 3.3. The OsPR10a is a potential candidate marker gene for radiation responses

From these two independent set of experimental results we found a candidate marker gene expression that showed the strongest dose-dependent response *(OsPR10a,* Figures 1D, 2, and 3, and [12]). Hence we propose that it could be utilized as a potential marker for ultra low-dose γ-radiation. For example, the *OsPR10a* promoter can be fused to fluorescent reporter genes to monitor the environmental radiation, either man-made or natural. Recently, it has been proposed that reference plants including wild grass (consisting of the well known cereal crops such as rice) should be considered as model test organisms in radiation studies by the International Commission on Radiological Protection [13]. Therefore, from the results presented here we can unravel the usefulness of a natural living organism (plant), in this case the grass model rice, for investigating ultra low-dose radiation responses. The only other report that comes close to this study is the effect of single whole-body X-radiation (acute dose) on clear induction of chromosomal inversion in the spleen and prostrate at ultra low-doses in a mammalian pKZ1 recombination mutagenesis mouse model [14].

## 4. Conclusions

The results arising from the present study using both natural CCS and in-house fabricated γ-ray sources provide evidence for ultra low-dose γ-radiation in causing perturbations in the multi-layered stress-associated biological processes in rice leaves. Further experiments are planned in the coming year to conduct high-throughput omic analysis post-γ -ray irradiation in both leaf segments *(in vitro)* and whole plants *(in planta/in vivo)* in short- and long-time exposures. This will help in a global profiling of radiation-induced molecular changes giving i) new candidate genes/proteins/metabolites for further study and ii) providing potential insight into the how ultra low-dose γ-ray triggers the defense/stress response. We wish to clarify one additional point in future studies. Do the molecular changes observed in leaves reflect a stress response leading to plant damage and/or death or are they indicative of an adaptation mechanism against the applied radiation? Pending these future studies, it is believed that the present study will lead to a new method to investigate radiation effects on gene: biological response to stimulation by ultra low-dose IR, which might also give some clue to the problem of by-stander effect of radiation. We also hope to rekindle the debate on understanding biological effects of ultra-, low-, and high-level radiation in living organisms, including our stationary life-sustaining “distant cousins” – the plants. It may be envisioned that rice will serve as a human model for investigating the responses to IR, due to conservation of genes/gene families in living organisms.

## Figures and Tables

**Table 1.** The γ-ray measurement was performed on 20 g contaminated soil before the decomposition of samples, using a Ge-detector, coupled to a multichannel analyzer. The available _90_Sr (β-ray emission spectra) in 1 g contaminated soil was measured by a low background β-ray spectrometer. CCS: Contaminated Chernobyl Soil −5.34 μGy/day; CON: Control Soil −0.49 μGy/day.

Radionuclide	Half-life	Energy (keV) [emission ratio]	Radioactivity [Bq/g-dry]
^241^Am *1)	432.2 year	59.54 (35.9%)	0.67 ± 0.013
^137^ Cs *1)	30.07year	661.7 (85.1%)	67.9 ± 0.024
^134^Cs *1)	2.065 year	604.7 (97.6%)	0.16 ± 0.003
		795.8 (85.5%)	0.18 ± 0.002
^90^Sr *2)	28.78 year	546 (100%)	3.18 ± 0.33

*1) Measurement by Gamma-ray spectrometer.

*2) Measurement by Beta-ray spectrometer.
